# Emotional Fertility Intention and its correlates in Ethiopia among married contraceptive user women: using linked community and health facility data from performance monitoring for action; a generalized ordered logistics regression modeling

**DOI:** 10.1186/s12889-024-19416-7

**Published:** 2024-07-30

**Authors:** Solomon Abrha Damtew, Mahari Yihdego Gidey, Fitsum Tariku Fantaye, Niguse Tadele Atnafu, Bezawork Ayele Kassa, Hailay Gebremichael Gebrekidan, Tariku Tesfaye Bekuma, Aynaw Amogne, Kelemua Mengesha Sene, Tariku Dejene Demissie

**Affiliations:** 1 Department of Epidemiology and Biostatistics, School of Public Health,, Wolaita Sodo University, Wolaita Sodo, Ethiopia; 2 PMA Project Addis Ababa University, Addis Ababa, Ethiopia; 3 FTF Research Consult, Addis Ababa, Ethiopia; 4 Adult Health Nursing, School of Nursing and Midwifery, Ababa University, Addis Ababa, Ethiopia; 5 University of Cape Town, Cape Town, South Africa; 6 Mekelle University, Mekelle, Ethiopia; 7 Wollega University, Nekemte, Ethiopia; 8 Kotebe Metropolitan University, Addis Ababa, Ethiopia; 9 College of Development Studies Addis, Addis Ababa University, Addis Ababa, Ethiopia

**Keywords:** Women health, Emotional fertility intentions, PMA Ethiopia, Linked data

## Abstract

**Background:**

Emotional fertility intention and couples communication are key during pregnancy and childbirth with simultaneous minimization of reproductive coercion. Intention to conceive is an integral part of the reproductive health (RH) right and can be considered as decision making on fertility, family wellbeing and the country’s population demographic dividend and composition. However, in low and middle income countries including Ethiopia where males dominance is culturally constructed and socially accepted, males took the lead in every decision making process. In the aforementioned context, women are less likely for their voices to be heard, hence, this study aimed at determining the level of womens´ emotional fertility readiness and its correlates. The finding provided actionable evidence for the ministry and developmental partners working on reproductive and womens´ health so as to be used as an action point to empower women in terms of their reproductive health right to have control over their fertility.

**Methods:**

Linked community and facility data with nationally representation from Performance Monitoring for Action (PMA Ethiopia) 2020 Survey Ethiopia except Tigray Region were used for this study. A total of 2,069 current and/or recent contraceptive user women of child bearing age who are currently married/living together as a partner were included in this analysis. Frequency was computed to describe the study participant’s characteristics. Generalized Ordered logistics regression modeling was employed to identify correlates of the hierarchical variation in women fertility intention if they became pregnant. Results were presented in the form of percentages and odds ratio with 95% Confidence Intervals. Candidate variables were selected using *p*-value of 0.25. Statistical significance was declared at *p*-value of 0.05.

**Results:**

The proportion of womens´ emotional fertility intention of feeling unhappiness was 48.73% (95%CI: 46.21%, 51.23%). On the contrary, 22.88%, 11.36% and 17.03% of them reported that they felt sort of happy, very happy and mixed feeling. An increase in age,10 and above years marriage duration, the type of decision maker for contraceptive use were found to increase the odds of women emotional fertility intention across the higher level categories by (AOR: 95% CI: 6.75 (3.11, 14.62) times higher among elder women aged 35 to 49 years, (AOR: 95% CI: 3.79 (1.72, 8.31) times higher for women with a 10 or more years of marriage duration; and 1.83 (1.03,3.24) times higher for women whose contraceptive use was decided by the health care provide alone. A higher birth order lowered the cumulative odds of womens´ emotional fertility intention symmetrically across the higher level categories by 86% (AOR: 95% CI: 0.14 (0.07, 0.29). Women who wanted to have additional child and whose nearest facility provided 5 or more methods had an increased odds of being in the higher level categories of women emotional fertility intention with disproportional association across the cumulative logit. Accordingly, women whose nearest health facility provided 5 or more methods had an 49% (AOR: 95%CI:1.49 (1.01, 2.19) increased likelihood of being in the mixed or happy category than being very/sort of unhappy category of the emotional fertility intention while the number of methods had no significant association with emotional fertility intention at higher cumulative logit: 1.34 (0.87,2.10). Those who wanted to have an additional child had a 3.16 (2.28, 4.36) higher odds to be in the mixed or happy category than being in unhappy category. Further, this tendency was even stronger at higher categories of emotional fertility intention: 4.83 (3.23, 7.23).

**Conclusion:**

Nearly one in two women reported being unhappy while 17.03% felt mixed emotion calling up on intended and spaced pregnancies by ensuring women reproductive and economic empowerment to empower women to have control over their fertility. Activities and efforts that promote intended and spaced pregnancies; and diversifying access to contraceptive methods in the nearest health facilities are likely to improve women emotional fertility intention; and activities that enable women to decide their contraceptive as well. The finding that health care provider decides on women current/recent contraceptive use calls for activities to improve quality of contraceptive use counseling to enable women to decide their contraceptive use by the themselves while the access of diversified methods in the nearby health facility create an opportunity for women to obtain the method they preferred to use and make them emotionally well. These activities are hoped to enable women to plan their fertility thereby increasing their emotional well-being. These activities and interventions need to be tailored across regions and need to be age sensitive.

## Background

Like physiologic changes during pregnancy and childbirth, this process is not free of stress and likely to affect the emotional well-being of the women. Emotional health and patient provider communication are key [1] during pregnancy and childbirth and involve the simultaneous minimization of reproductive coercion [2]. Spéder & Kapitány stated that happier men and women prefer to became parents sooner arguing that there is a stronger link between intention and behavior towards fertility [3, 4]. Moreover, couples´ happiness and emotion determines having a child particularly women's happiness and perceived fertility emotion matters more on the decision making process towards having subsequent child [5]. Another evidence showed that fertility intention and behavior has a closer link [6] indicating that fertility emotion and behavior influences the demographic dividend of countries. However, the emotional aspect of fertility intention among women has been usually overlooked.

Intentions to conceive is an integral part of women reproductive health right and can be considered as decision making on fertility, family well-being and the country’s population demographic dividend and composition [7–11]. However, in low and middle income countries including Ethiopia where males dominance is culturally constructed and socially accepted, males take the lead in every decision making process for the families: ranging from household level decision to determing the size of the family along with the family’s health service use. In low and middle income countries fertility desired is determined by the husband [12]. Males dominance in decision making is practiced as far sexual and reproductive health concerned [12–15]. Further evidence also showed that discordance in fertility desire among couples [16]. In such a context of socially constructed and culturally accepted males dominance, women voices may not have been heard.

As a result of this males dominance on fertility control, women are likely to held negative and unpleasant feeling and emotion when every they thought of becaming pregnant or learned that they are pregnant [14]. Due to the unproportional huge burden of raising children experienced by most women in developing countries coupled with family leading burden including domestic chores, women tend to held negative emotion for every additional pregnancy and child they are going to bear. Unless evidence is generated and timelyaction is taken such unpleasant feeling exacerbates the increasing fertility rate and population dynamics being in midst of rapid population growth [3, 12, 17]. Moreover, the success of ending poverty in all its forms everywhere which is clearly articulated in the sustainable development goal 1 (SDG_1) has been precipitated by Countries' rapid population growth through putting more pressure on already depleted resources, thereby creating a greater challenge to ensure sustainable development goals (SDG) [18]. Studies show that if the current population increases by 40% is not controlled and be in the line with the country’s economic development, the economy, food production, general environment, and global climate will be severely affected [19, 20].

In Ethiopia, although many activities have been done to control the rapid population growth and reduce the average number of births per woman [21–23], in the last decade, it has not been feasible to achieve the desired level of change as was planned and intended in the national health sector transformation plane (HSTP) and the national reproductive health (RH) strategies [24–26]. Both the annual population growth and fertility rate remain high at 2.7 and 4.6, respectively [27]. A lack of emotional readiness for fertility and child bearing is likely to contribute for such sustained higher fertility rate and subsequent health and economic effects [7, 28].

Fertility desire can be influenced by several factors that operate at the societal and individual levels. At the societal level, the desire for fertility is often driven by social and cultural pressures and the desire to maintain the stability of society [29, 30], such as strong cultural preferences for large families [29, 31] and the desire for boys over girls [32–35]. Such cultural and social element along with sex preference precipitated the emotional aspect of fertility intention. At the individual level, characteristics including age [36, 37], number of living children [38, 39], marital status, wealth, education level [40–44], and place of residence [45–47] and sociodemographic characteristics of the women [40, 41] are associated with fertility desire. Although numerous factors have been documented to influence fertility desires in different parts of the world, there is a relative dearth of literature in Ethiopia, where significant proportion of women who have several children still desire to have more children [40, 48]. Moreover, there is dearth of evidence on women emotional fertility intention, what do they feel if they became pregnant among women in general and those who are current and/or recent contraceptive users in particular [49].

As per the World Health Organization definition of health, this study attempted to address the mental/emotional and social aspect of health since pregnancy and child birth is a communal event in Ethiopian and in other low and middle income countries [50]. To this end, emotional aspect of the fertility, how would they feel if they get pregnant is less explored in Ethiopia and there is a dearth of evidence. Womens´ fertility emotion might be related with religion and culturally acceptability of large families [51]. Hence, determining the degree of women emotion towards bearing additional child and identifying its correlates contributing for such hierarchical variation among married contraceptive user women is very critical to improve women and newborn health out comes and contributes its share in halting the rapid population growth. This could provide actionable evidence to mitigate the huge fertility surge thereby empowering women over their fertility in particular along with improved health of newborns and families.

## Methods and data sources

### Study design and population and sample size

Linked community and health facility cross sectional data from the Performance Monitoring for Action (PMA) 2020 Survey were used for this study. All women of child bearing age from the selected randomly 35 households per EA were interviewed for the female questionnaire, hence a total of 2,069 current and/or recent modern contraceptive user women of child bearing age who are married/living together as a partner with a man were included in this analysis. The overall sample size and cell sample size adequacy was checked and found adequate to generating unbiased estimates for the women emotional fertility intention.

### Data collection and field work

This study is a further analysis of nationally representative linked community and facility level data from Ethiopia Performance Monitoring for Action (PMA Ethiopia) 2020 Survey except Tigray Region. Frequency was computed to describe the study participant’s characteristics [49]. A two stage stratified cluster sampling method was used to select enumeration areas. Following EA delineation complete listing of all residential households was conducted in the selected enumeration areas with the aim to prepare fresh sampling frame. The size of the households varied form one enumeration area to another and between urban and rural areas as well and failing within the average number of households allocated per enumeration area while preparing EA maps by Ethiopian Statistical Services for rural and urban enumeration areas. Then, supervisors selected 35 households per enumeration area using simple random sampling technique by random number generator application after verifying the correctness of the sample frame.

It was executed by Addis Ababa University’s School of Public Health in collaborative efforts with the Ethiopian Public Health Association with assistance from the Federal Ministry of Health, Central Statistical Agency, Bill & Melinda Gates Institute for Population and Reproductive Health (Johns Hopkins Bloomberg School of Public Health).

The main sample units or enumeration areas (EAs) were chosen using the frame to Ethiopia Population and Housing Census (PHC), which was performed in 2019 by the Ethiopia Ethiopian Statistical Services. A total of 213 EA were chosen in the first stage, with Independent selection in each sample stratum and a probability proportional to EA size. The protocol of PMA Ethiopia contains all the details on sample design and selection methods. More detail in the details on sampling design and selection procedures and field work implementation was described well elsewhere [49].

### Variables

#### Outcome variable

Women emotional fertility intention was the main outcome variable. It was measured by a single Likert scale question with 5 scales as shown below (Table 1). Women were asked if you get pregnant now how would you feel with the five response options shown in the table below. For the seek of getting a meaning out this specific scale of measurement, the scale was reverse coded that the very happy category received the largest value on the scale of measurement and the very unhappy category assigned the smallest value in the scale since women were asked in the reverse order and the question is asked in a positive way.

**Table 1:** Items used to measure women emotional fertility Intention, Performance Monitoring for Action 2020 Linked Community and Facility Data

Emotional Fertility Desire of women If you got pregnant now, how would you feel?	**Variable**	**Question Items and Responses**	**Recode Categories(Reverse coded hierarchical order)**
	Feeling if you got pregnant now Women were asked if you get pregnant now how you would you	1. Very Happy	1. Very unhappy 2. A sort of unhappy 3. Mixed happy and unhappy 4. A sort of happy5. Very happy
		2. Sort of happy	
		3. Mixed happy and unhappy	
		4. Sort of unhappy	
		5. Very Happy **Reverse code:** For descriptive: five categories! recode preg_now_reaction (-88 -99=.) (1=5 "Very Happy") (2=4 "Sort of Happy") (3=3 "Mixed Un/Happy") (4=2 "Sort of unhappy") (5=1 "Very Unhappy"),gen(preg_now_reaction_Rev5) ta preg_now_reaction_Rev5 for regression: recode preg_now_reaction (-88 -99=.) (1=5 "Very Happy") (2=4 "Sort of Happy") (3=3 "Mixed Un/Happy") (4=2 "Sort of unhappy") (5=1 "Very Unhappy"),gen(preg_now_reaction_Rev5) ta preg_now_reaction_Rev5 recode preg_now_reaction_Rev5 (1 2=1 "Very /sort un/Happy") (3=2 "Mixed Un/Happy") (4 5=2 "very/Sort of happy"),gen(preg_now_reaction_Rev3) ta preg_now_reaction_Rev3 //order// recode preg_now_reaction_Rev5 (1 2=0 "unHappy") (3=1 "Mixed") (4 5=2 "Happy"),gen(preg_now_reaction_Rev333)	3 Category Reverse coded 4 5 = 2 Very happy and sort of happy 3=1 Mixed happy and unhappy 1 2 = 0 Very unhappy and sort of unhappy

Note the five scale ordinal variable was condensed to three level ordinal outcome due to cell sample issue for some independent variables

#### Independent variables

Independent variables were classified into individual-level variables and enumeration area-level Variables. Individual-level independent variables were further categorized into sociodemographic/economic characteristics variables, parity and other RH characteristics, knowledge of contraceptive related characteristics and husband related characteristics.

Group or enumeration area (EA) level variables included two integral variables namely, region and place of residence while three derived EA level variables: EA level wealth derived from the household level wealth, the proportion of women who completed secondary education or above per EA and her husband/partner´s who completed the same educational level per EA were derived from the respective women and husband/partner´s educational status. “Region” was grouped into five categories 3 = Amhara, 4 = Oromia, 7 = SNNPRs and 10 = Addis Ababa city administration and other Regions. Place of residence follows the default urban/rural classification.

The data used for this study can be obtained upon request from PMA, the details are mentioned in elsewhere [49], and as can stated in the declaration part in this manuscript and the manuscript tracking system: the datasets used to produce estimates for this study were publicly available from the PMA website. https://www.pmadata.org/data/request-access-datasets, the publicly available versions of the questionnaire were provided with data up the request and uploaded in the manuscript tracking system as supplementary file.

### Analysis and measurement

A linked community and health facility (SDP) data; namely the 2020 PMA cross sectional community and SDP data were used for this piece of analysis. The Household/Female data were linked with the SDP data using the household and SDP GPs coordinate points based on the principle of the nearest facility. The following key concepts and steps were employed to like the community data with the service facility point (SDP) data sets:

The date sets were merged Using GPS coordinates, which is displaced for the analysis purpose.

- All SDPs and all women have GPS coordinates, which were displaced for the analysis purpose.
- The household/female and SDP datasets were merged and, the distance between each woman and each SDP was calcualted
- The observations that the authors need to consider in the this analysis were kept.
- Steps to link
- Identify the number of SDPs in the dataset (x=#SDPs), x=744
- Create linking variable (gen linkvar=_n)
- Expand the female sample to include x number of observations per female (expand x)
- Create same linking variable (bysort FQmetainstanceID: gen linkvar=_n)
- Merge datasets m:1 using linkvar

Link variable was created using displaced GPs coordinates to link the community and facility data sets. Each enumeration area was matched with the nearest Service delivery point (SDP). First the required variables were kept from the SDP data set.

Data were analyzed using STATA v16: Copyright 1985–2019 StataCorp LLC Statistics/Data Analysis StataCorp 4905 Lakeway Drive, Special Edition College Station, Texas 77,845 USA, 800-STATA-PC http://www.stata.com, 979–696-4600 stata@stata.com, 979–696-4601 (fax). Single-user Stata perpetual license with Serial number: 401606348636.

Frequencies and percentages were computed to characterize the study population. Chi-square test statistics was computed to check cell sample size adequacy and the sample size was found to be adequate to provide unbiased estimates for emotional fertility intention among contraceptive user married women or those currently living with a man as a partner. Exploratory data analysis was run for data cleaning thereby checking item nonresponse rate for every variable and don’t know response which were subsquently excluded from the analysis. Following this variable were recoded to create biologicaly plausible categories along with checking distribution of the recoded variables using mean and proportion. Composite variable was created; namely, source heard about contraceptive methods and knowledge on contraceptive methods. Integral, facility related factors/group level variables such as distance from the nearest health facility, type of the nearest facility, method type provided by the nearest health facility and method stock out in the nearest health facility were considered in this analysis.

Generalized Ordinal logistics regression (GOLR), (partial proportional odds model) was used to identify important correlates of women emotional fertility intentions, what did they felt if they became pregnant by then. At bivariate analysis a p value cut of 0.25 [52] was used to select candidate variable for multivariable generalized ordered logistics regression analysis. Results were presented in the form of percentage, and odds ratio with 95% CI. Significance was declared at a significance level of 0.05. Results were reported based on weighted count.

Unlike the conservative ordered logistics regression the GOLR is relaxes the assumption of proportional odds for some variables [53]. Since practically the parallel line assumption is most of the time violated using the generalized ordered model entertains this practical phenomenon. It can be less restrictive than proportional odds models and more parsimonious than methods that ignore the ordering of categories altogether [53]. Hence, generalized ordered logistic regression modeling was run to determine the correlates of the women emotional fertility intention [53, 54].

Given an ordinal outcome variable, the potential model to be fit to the data is the ordered logit model. In order for the use of the ordered logit model to be valid, the assumptions that the effect of the independent variables on the odds ratio is constant across all the cutoff points between the categories should be met. This assumptions of the model are referred to as the parallel lines or parallel regressions assumptions. The Brant test is a commonly used approach to assess whether the proportional odds assumption has been violated. During the analysis, the Brant test yielded that the assumption of proportional odds was violated for ‘*fertility desire*’ and ‘*facility type’*. Consequently, generalized ordered logit model, an alternative to the ordered logit model, was fit to the data. The generalized ordered logit model is an alternative approach that relaxes the proportional odds assumption. The effect of the explanatory variables on the odds of being in a higher category versus a lower category can vary across the different cut-off points of the ordinal outcome. This flexibility of the model comes with the increased number of parameters estimated than the conventional ordered logit model [53].

### Data quality management and control

Data completeness for variables and items for creating composite variables were checked by exploratory data analysis following which any item nonresponse was excluded from the analysis. Frequency run to exclude responses with do not know (DNK) and no response (NR). Based on weighted count, of the total 2,113 current and/or recent contraceptive users who are married/or living as a partner and 2,098 women gave complete response for the fertility emotion variable (14 responded do not know and item non response for one respondent).

PMA Ethiopia used of standard and pretested tool, intensive training with mock interviews for resident enumerators, close supervisor during filed work, timely progress report and correction, 10% random check were some of the modalities used to maintain the quality of the collected data, the detail is reported somewhere else [49].

### Ethical consideration

This study involved a secondary analysis of de_identified data from the PMA Ethiopia. The PMA Ethiopia survey was conducted strictly under the ethical rules and regulations of world health organization and IIRB of Ethiopian Health and Nutrition Research Institute (EHNRI). Informed consent was obtained from respondents during the data collection process of PMA Ethiopia on data collection on Nov 2020 to Jan 2021. PMA surrey has been also conducted after obtained ethical approval from the College of Health Sciences at Addis Ababa University and Bloomberg School of Public Health at Johns Hopkins University in Baltimore, USA. PMA_ETH Publicly available Cohort one baseline and six weeks postpartum datasets were accessed after submitting a concept note for this piece of specific work form the PMA data cloud server archive via. https://www.pmadata.org/data/request-access-datasets.

Since this study involves analysis of already collected secondary data there is no need to consent, rather, concept note was submitted to get permission for data use.

“Minors less than 15 years as per the law were not included in this study. Informed verbal consent was take from study participants.” Moreover, women of reproductive age group or women child bearing age were include in the study. The survey includes topics on related on family planning, sexual history and other reproductive health issues which is declared are right of women by international declarations and as supported by evidence: Rimon JGII, Tsui AO. Regaining momentum in family planning. Global Health Science Practice. 2018; 6(4):626–628. 10.9745/GHSP-D-18-00483 2018. Such topics are waived to be directly asked for the women themselves about their sexual and reproductive issues. Moreover, standard surveys including Demographic and health surveys include women of child bearing age as this study did.

## Result

### Magnitude of women emotional futility intention among married contraceptive user women evidence from PMA 2020 CS survey

**Fig. 1:**
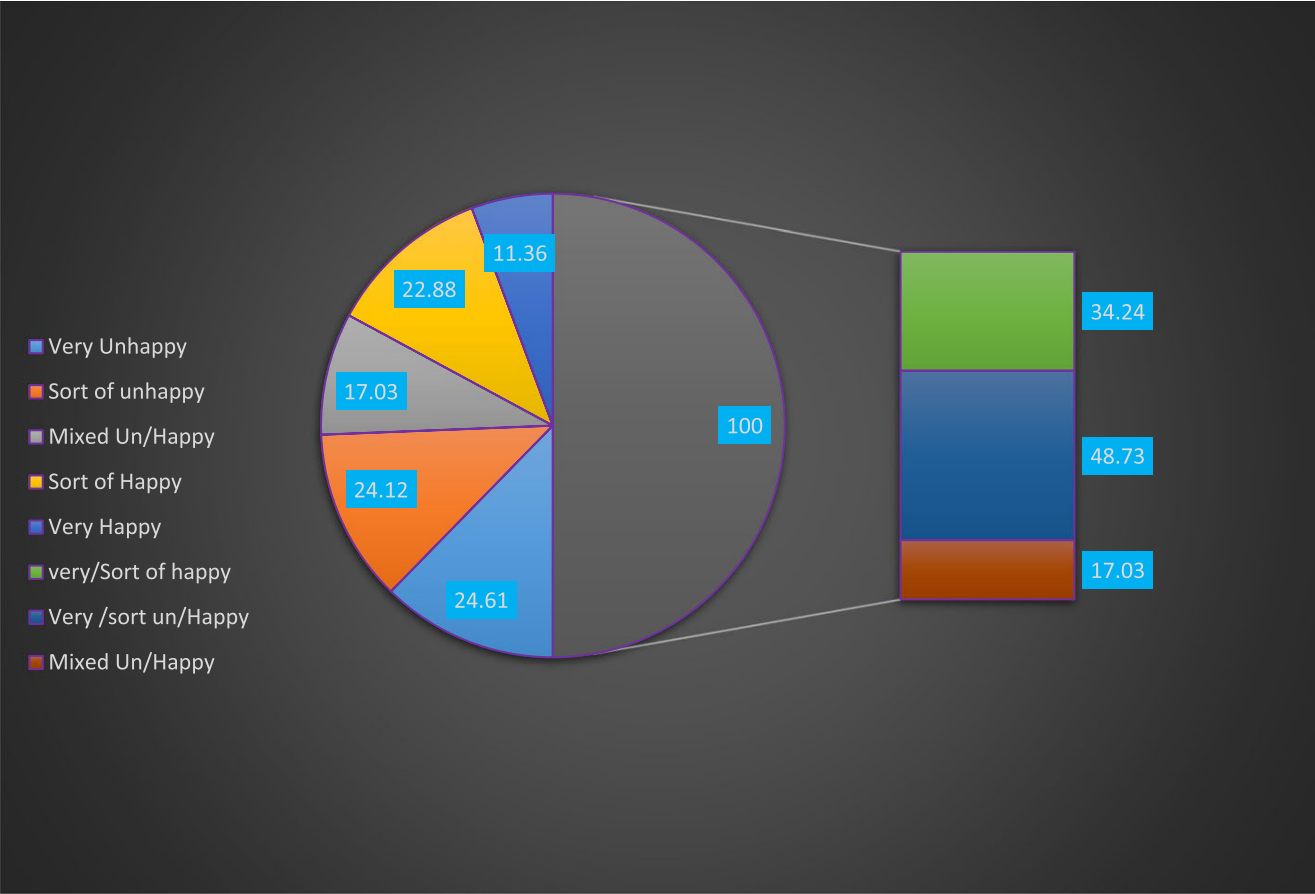
Emotional fertlity intention among married contraceptive user women

The proportion of women emotional fertility intention of being unhappy was nearly one in two; 48.73% (95% CI: 46.21%, 51.23%). Likewise, 17.03% (95% CI:15.26%, 18.96%) felt mixed feeling and one third, 33.24% (95% CI:31.89%, 36.69%) felt very/sort of happy if they became pregnant (Fig. 1).

### Distribution of women emotional fertility intention among married contraceptive user women, evidence from performance monitoring for action 2020 linked community and facility data

Among women in the age category 35 to 49 years 29.67%, 20.10%, 17.44%, 21.39% and 11.40% reported that they felt very unhappy, sort of un happy, mixedfeeling, sort of happy and very happy respectively when asked how do you felt if you became pregnant. Similarly, of women in the secondary education or above category, the proportion of those who felt very unhappy, sort of un happy, mixed feeling, sort of happy and very happy was 15.77%, 21.99%,19.65%, 28.31% and 14.28% respectively.

This same figure for women belonging in the households with middle wealth quintile was 32.96%, 20.89%, 19.49%, 17.0% and 9.67% for the respective feeling categories. For residents of Amhara region, 20.27%, 21.22%, 20.52%, 27.20% and 10.77% of women were very unhappy, sort of unhappy, mixed feeling, sort of happy an very happy respectively. Likewise, for rural residents, this similar figure for the respective women emotional fertility intention categories was 26.02%, 25.45%, 16.11%, 21.28% and 11.13% (Table 2).

**Table 2:** Distribution of Women Emotional Fertility Intention among Married Contraceptive User Women, by Socio-demographic Characteristics variable Evidence from Performance Monitoring for Action 2020 Linked Community and Facility Data *n* = 2069

		Very Unhappy	%	Sort of unhappy	%	Mixed Feeling	%	Sort of Happy	%	Very Happy	%	Total
Age Category	15–19 years	27	20.99	46	35.44	21	15.87	25	19.06	11	8.63	130
	20–24 years	104	24.18	109	25.33	68	15.69	108	25.09	42	9.71	431
	25–29 years	116	22.47	128	24.68	104	20.13	120	23.13	50	9.59	517
	30–34 years	97	22.24	105	23.97	64	14.52	103	23.42	70	15.86	438
	35–49 years	164	29.67	111	20.10	96	17.44	118	21.39	63	11.40	553
	Total	509	24.61	499	24.12	352	17.03	473	22.88	235	11.36	2069
Education Status	No Education	207	28.88	165	22.96	111	15.41	159	22.16	76	10.59	718
	Primary	223	26.16	225	26.34	144	16.87	173	20.32	88	10.31	853
	Secondary Plus	78	15.77	109	21.99	98	19.65	141	28.31	71	14.28	497
	Total	509	24.61	499	24.12	352	17.03	473	22.88	235	11.36	2069
Wealth Index	Lowest quintile	79	23.20	92	26.88	45	13.30	89	26.00	36	10.62	342
	Lower quintile	109	26.71	107	26.23	71	17.41	84	20.58	37	9.06	407
	Middle quintile	131	32.96	83	20.89	78	19.49	68	17.00	39	9.67	399
	Higher quintile	100	24.93	104	25.95	58	14.57	91	22.83	47	11.72	400
	Highest quintile	90	17.25	113	21.73	100	19.19	142	27.16	76	14.67	521
	Total	509	24.61	499	24.12	352	17.03	473	22.88	235	11.36	2069
Region	Other Regions	9	15.27	13	23.24	10	17.50	15	26.07	10	17.91	57
	Amhara	125	20.27	130	21.22	126	20.52	167	27.20	66	10.77	615
	Oromaia	243	26.79	223	24.62	137	15.13	184	20.33	119	13.13	905
	SNNPR */*	114	30.21	109	28.82	54	14.42	75	19.74	26	6.82	377
	Addis_Ababa	19	16.78	24	20.72	25	21.61	33	28.57	14	12.32	114
	Total	509	24.61	499	24.12	352	17.03	473	22.88	235	11.36	2069
Place of residence	URBAN	140	21.53	138	21.23	124	19.03	172	26.34	77	11.86	652
	RURAL	369	26.02	361	25.45	228	16.11	302	21.28	158	11.13	1417
	Total	509	24.61	499	24.12	352	17.03	473	22.88	235	11.36	2069
Family Size	1 to 3 members	88	16.24	124	22.73	94	17.25	161	29.61	77	14.17	543
	4 to 5 members	218	25.90	204	24.25	159	18.84	171	20.29	90	10.71	842
	6 to 16 members	203	29.66	171	25.06	100	14.62	142	20.72	68	9.94	684
	Total	509	24.61	499	24.12	352	17.03	473	22.88	235	11.36	2069
Birth order	No child	25	14.02	38	21.02	21	11.69	52	28.96	44	24.31	179
	1 child	80	17.11	113	24.20	92	19.68	134	28.59	49	10.41	468
	2 Children	105	24.56	106	24.88	92	21.61	79	18.44	45	10.51	426
	3 children	73	25.13	82	28.05	39	13.49	66	22.75	31	10.58	292
	4 + children	226	32.09	160	22.77	108	15.32	143	20.27	67	9.54	704
	Total	509	24.61	499	24.12	352	17.03	473	22.88	235	11.36	2069
Marriage Type	Monogamy	467	24.20	467	24.23	328	17.02	456	23.64	210	10.90	1928
	Polygamy	41	29.85	31	22.78	24	17.65	17	12.80	23	16.92	136
	Total	507	24.58	498	24.13	352	17.07	473	22.93	233	11.30	2064

Among those women who reported a family size of 1 to 3, the proportion of feeling very unhappy and sort of un happy was 16.24% and 22.73% while mixed feeling, sort of happy and very happy accounted for 17.325%, 29.61% and 14.17% respectively (Table 2).

Concerning contraceptive knowledge among those who reported good contraceptive knowledge, 25.0%, 23.20%, 17.03%, 22.46% and 12.31% were very unhappy, sort of unhappy, mixed feeling, sort of happy and very happy respectively. For those who have heard about contraceptive from at least one source in the last 12 month; this respective figure was 23.14%, 24.07%, 16.76%, 22.42% and 13.61%. Similarly, among those who reported that they have obtained their desired method, 24.68% were very unhappy, 23.95% felt sort of unhappy, 17.43% reported mixed feeling; and 22.67% and 11.27% felt a sort of happy and very happy respectively. This same figure for those who did not told about other methods stood 22.85%, 24.89%, 17.27%, 23.21% and 11.78% respectively for the respective fertility emotional categories (Table 3).

**Table 3:** Distribution of Women Emotional Fertility Intention among Married Contraceptive User Women, Contraceptive use Characteristics variables Evidence from Performance Monitoring for Action 2020 Linked Community and Facility Data *n* = 2069

		Very Unhappy	%	Sort of unhappy	%	Mixed Feeling	%	Sort of Happy	%	Very Happy	%	Total
Contraceptive Methods Knowledge	Poor Knowledge	94	23.02	114	27.88	69	17.01	100	24.58	31	7.51	408
	Good Knowledge	415	25.00	385	23.20	283	17.03	373	22.46	204	12.31	1661
	Total	509	24.61	499	24.12	352	17.03	473	22.88	235	11.36	2069
Source of Contraceptive Information	No Information	310	25.53	294	24.21	210	17.25	282	23.18	119	9.83	1215
	At least one source	196	23.14	204	24.07	142	16.76	190	22.42	115	13.61	847
	Total	506	24.55	498	24.15	352	17.05	472	22.87	235	11.38	2062
Obtaining Desired Method	No	56	24.00	59	25.48	32	13.89	57	24.52	28	12.11	233
	Yes	453	24.68	440	23.95	320	17.43	416	22.67	207	11.27	1836
	Total	509	24.61	499	24.12	352	17.03	473	22.88	235	11.36	2069
Fertility Desire	No More Another child	220	37.16	165	27.76	116	19.53	72	12.10	20	3.44	593
	Have Another child	289	19.58	333	22.58	237	16.04	402	27.23	215	14.56	1475
	Total	509	24.62	498	24.07	352	17.04	473	22.89	235	11.37	2068
Facility Visit last 12 m	No	176	26.16	141	20.98	123	18.21	147	21.82	86	12.83	674
	Yes	333	23.86	358	25.64	230	16.46	326	23.39	149	10.66	1395
	Total	509	24.61	499	24.12	352	17.03	473	22.88	235	11.36	2069
Type of method used	Short acting	309	23.88	317	24.52	236	18.26	288	22.28	143	11.06	1295
	Long acting	146	24.33	138	23.06	103	17.21	145	24.26	67	11.14	598
	Total	455	24.02	455	24.06	339	17.93	434	22.91	210	11.09	1893
Told Other Methods	No	258	22.85	281	24.89	195	17.27	262	23.21	133	11.78	1129
	Yes	216	22.31	214	22.11	179	18.49	235	24.28	124	12.81	968
	Total	474	22.60	495	23.61	374	17.84	497	23.70	257	12.26	2097

Among those who reported that they did not have discussed about contraceptive methods, 25.35%, 23.45%, 15.19%, 22.78% and 13.22% of them emotionally felt very unhappy, sort of unhappy, mixed feeling, sort of happy and very happy respectively. Similarly, among those women who reported that their husband/partner forced them to became pregnant 23.89%,16.10%, 12.9%,24.86% and 22.27% felt unhappy, sort of unhappy, mixed happy and unhappy, sort of happy and very happy respectively (Table 4).

**Table 4:** Distribution of Women Emotional Fertility Intention among Married Contraceptive User Women, by Partner Characteristics Variables Evidence from Performance Monitoring for Action 2020 Linked Community and Facility Data *n* = 2069

		Very Unhappy	%	Sort of unhappy	%	Mixed Feeling	%	Sort of Happy	%	Very Happy	%	Total
Discussion with Hus before use	No	108	25.35	100	23.45	65	15.19	97	22.78	56	13.22	427
	Yes	401	24.41	399	24.29	288	17.51	376	22.90	179	10.88	1642
	Total	509	24.61	499	24.12	352	17.03	473	22.88	235	11.36	2069
Husband forced to became Pregnant	Not Force	480	24.83	473	24.49	330	17.07	442	22.87	208	10.74	1933
	Force Preg	26	23.89	18	16.10	14	12.89	27	24.86	24	22.27	109
	Total	506	24.78	491	24.04	344	16.85	469	22.98	232	11.35	2042
Marriage duration	< 1 year	14	16.92	25	29.70	10	11.89	19	22.79	16	18.69	85
	1 to 9 years	203	22.79	223	25.04	164	18.40	220	24.69	81	9.08	891
	10 and above years	290	26.75	249	23.02	178	16.43	229	21.18	137	12.63	1084
	Total	507	24.63	498	24.17	352	17.10	469	22.76	234	11.34	2059
Contraceptive Use Decision Making	Women Alone	267	25.28	259	24.57	191	18.05	224	21.19	115	10.92	1056
	Provider	17	19.63	19	20.82	12	13.59	23	25.59	18	20.37	89
	Partner	16	28.86	11	19.36	8	13.99	14	25.69	7	12.10	54
	Women and Provide	23	21.84	33	31.24	16	14.75	20	18.56	15	13.60	107
	Women and Partner	186	24.38	177	23.26	126	16.58	192	25.19	81	10.58	762
	Total	509	24.62	499	24.13	352	17.04	472	22.84	235	11.37	2068
Age at first Sex	10 to 15 years	186	27.74	151	22.54	122	18.12	154	22.92	58	8.68	671
	16 to 20 years	273	24.71	291	26.35	166	14.97	246	22.20	130	11.77	1106
	21 and 36 years	48	17.28	54	19.36	61	21.92	70	25.10	45	16.33	278
	Total	507	24.69	496	24.16	348	16.94	469	22.83	234	11.38	2055
Partner Know using FP	No	45	25.57	44	25.00	24	13.64	36	20.45	27	15.34	176
	Yes	429	22.32	452	23.52	350	18.21	461	23.99	230	11.97	1922
	Total	474	22.59	496	23.64	374	17.83	497	23.69	257	12.25	2098
Husband/Partner Feeling Contraceptive Use	He disapp Does not care_	101	24.97	99	24.47	61	15.19	82	20.21	61	15.15	404
	He is oK with it	408	24.52	400	24.04	291	17.48	392	23.52	174	10.45	1665
	Total	509	24.61	499	24.12	352	17.03	473	22.88	235	11.36	2069

The respective emotional fertility intention among those who reported using long acting and/or permanent was 24.33%, 23.06%, and 17.21%, 24.26% and 11.14% (Table 3). Among those who stayed more than 10 years in marriage: 26.75%, 23.02%, 16.43%, 21.18% and 12.63% were unhappy, sort of unhappy, mixedfeeling, sort of happy and very happy respectively (Table 4).

Among those who decided their contraceptive use by themselves 25.28%, 24.57%, 18.05%, 21.19% and 10.92% were unhappy, sort of unhappy, mixed feeling, sort of happy and very happy respectively. The proportion of women with emotional fertility intention of being very unhappy, sort of unhappy, mixed feeling , sort of, happy and very happy was 27.74%, 22.54%, 18.12%, 22.92% and 8.68% be respectively among those reported that they started their first sexual intercourse within the age category 10 to 15 years (Table 4).

The proportion of women with emotional fertility intention of being very unhappy, sort of unhappy, mixed feeling, sort of happy and very happy was 25.57%, 25.0%, 13.64%, 20.45% and 15.34% respectively among who reported that their husband/partner did not knew that they are using or have been using contraceptive methods (Table 3). Among those who reported that their husband/partner was ok with their contraceptive use the proportion of women with emotional fertility intention of being very unhappy, sort of unhappy, mixed feeling, sort of happy and very happy was, 24.52%, 24.04%, 17.48%, 23.52% and 10.45% respectively (Table 4). Among those who gave birth of 4 more children 32.09%, 22.77%,15.32%, 20.27% and 9.54% were very unhappy, sort of unhappy, mixed feeling, sort of happy and very happy respectively if they would pregame now (Table 2).

Regarding distance from health facility, the proportion of women with emotional fertility intention of being very unhappy, sort of unhappy, mixed feeling, sort of happy and very happy was 32.48%, 23.78%, 18.78%, 16.88% and 8.07% respectively among those who reported there are living in area at 2 km and above from the nearest health facility. For women who reported that their nearest facility type is hospital: 17.5%, 18.58%, 21.54%, 27.13% and 15.25% felt being very unhappy, sort of unhappy, mixed feeling, sort of happy and very happy respectively. Likewise, 28.37%, 23.82%. 16.68%, 20.31% and 10.82% reported being very unhappy, sort of unhappy, mixed feeling, sort of happy and very happy if they got pregnant among women whose nearest health facility which is serving them experienced stock out of at least one method. The proportion of women who felt very unhappy, a sort of unhappy, mixed feeling, a sort of happy and very happy was 24.58%,21.89%,17.99%, 23,57% and 11.97% respectively among those whose nearest health facility provided 5 or more contraceptive methods (Table 5).

**Table 5:** Distribution of Women Emotional Fertility Intention among Married Contraceptive User Women, by SDP Characteristics Variables Evidence from Performance Monitoring for Action 2020 Linked Community and Facility Data *n* = 2069

		Very Unhappy	%	Sort of unhappy	%	Mixed Feeling	%	Sort of Happy	%	Very Happy	%	Total
Distance to HF	0 to 1 km	193	19.98	230	23.74	172	17.79	252	26.06	120	12.43	968
	1 to 2 km	132	24.67	135	25.17	74	13.80	126	23.45	69	12.92	536
	2 km and above	183	32.48	134	23.78	106	18.79	95	16.88	46	8.07	565
	Total	509	24.61	499	24.12	352	17.03	473	22.88	235	11.36	2069
Type of nearest Health Facility	Hospital	7	17.50	7	18.58	8	21.54	10	27.13	6	15.25	38
	Health center	137	27.93	100	20.46	94	19.25	110	22.55	48	9.80	490
	Health post	280	23.35	314	26.22	196	16.40	272	22.74	135	11.30	1198
	Health clinic	86	25.05	78	22.63	53	15.57	80	23.37	46	13.38	344
	Total	509	24.61	499	24.12	352	17.03	473	22.88	235	11.36	2069
Method Provided at nearest health	Provide_4methods	199	24.66	223	27.60	125	15.53	176	21.80	84	10.41	808
	Provide_5 + Methods	310	24.58	276	21.89	227	17.99	297	23.57	151	11.97	1261
	Total	509	24.61	499	24.12	352	17.03	473	22.88	235	11.36	2069
SDP Stock Out Experience	No Stock out	267	21.98	296	24.33	210	17.27	300	24.68	143	11.74	1217
	At least One Stock out	242	28.37	203	23.82	142	16.68	173	20.31	92	10.82	852
	Total	509	24.61	499	24.12	352	17.03	473	22.88	235	11.36	2069

*/* former Southern Nations, Nationalities and peoples Region

### Correlates of Women Emotional Fertility Intention among Married contraceptive user women, evidence from performance monitoring for action 2020 linked community and facility data

This study investigated factors contributing for the hierarchal variation of women emotional fertility intention measured with 5 items ordinal scale and later categorized in to three ordinal categories (very unhappy, mixed feeling and happy) using generalized ordered regression modeling which gave us two cumulative logit for the two higher level categories (The re categorization was needed for the seek of cell sample adequacy). Fertility desire and number of contraceptive methods provided in the nearest health facility were found to violate the proportional odds assumption, hence, generalized ordered logistics regression run. The generalized logit model is also called cumulative logit as it determines the cumulative probability of being in different combination of the higher level categories of the outcome variable, in this case, women fertility intention feeling result was reported (Table 6).

**Table 6:** Generalized Ordered Logistics Modeling for covariates of hierarchical variation in Women Emotional Fertility Intention among Married Contraceptive User Women, Performance Monitoring for Action 2020 Linked Community and Facility Data

Variables		Unhappy AOR	Mixed Feeling AOR^+^
	15 to 19 years	1	
Age Category	20–24 years	2.32 (1.24,4.34)**	
	25–29 years	3.73 (1.91,7.32)***	
	30–34 years	5.41 (2.48,11.79)***	
	35–49 years	6.75 (3.11,14.62)***	
Educatioanal Status	No Education	1	
	Primary	0.95 (0.67,1.33)	
	Secondary Plus	1.31 (0.77,2.22)	
Welath Index	Lowest Quintile	1	
	Lower quintile	0.84 (0.51,1.37)	
	Middle quintile	0.70 (0.42,1.17)	
	Higher quintile	0.63 (0.34,1.17)	
	Highest quintile	1.08(0.53,2.24)	
Religion	Orthodox	1	
	Protestant	1.03. (0.71,1.47)	
	Muslim	0.90 (0.58,1.39)	
Residence	Urban		
	RURAL	1.09 (0.70,1.71)	
Family Size	1 to3 members	1	
	4 to 5 members	0.86 (0.62,1.19)	
	6 to 16 members	0.81 (0.52,1.26)	
Marriage type	Monogamy	1	
	Polygamy	1.04 (0.63,1.72)	
Contraceptive Knowledge	Poor Knowledge	1	
	Good Knowledge	0.95 (0.69,1.32)	
Contraceptive Information	No Information	1	
	At least one source	1.05 (0.80,1.38)	
Desired Method obtained	No	1	
	Yes	0.93 (0.57,1.51)	
	No More_Another Child	1	
Fertility Desire	Intended_Have_Another_child	3.16 (2.28,4.36)***	4.83 (3.23,7.23)***
visited_a_facility	No	1	
	Yes	0.88 (0.68,1.15)	
Told other Methods	No		
	Yes	1.11 (0.85,1.37)	
Contraceptive Method type	Short Acting	1	
	long acting	1.05 (0.81,1.16)	
Marriage Duration	Less than 1 year	1	
	1 to 9 years	2.28 (1.16.4.51)*	
	10 and above years	3.79 (1.72,8.31)**	
Contraceptive Use Decision Maker	Women Alone		
	Provider	1.83 (1.03,3.24)*	
	Partner	1.31 (0.62,2.75)	
	Women and Provide	1.24 (0.67,2.30)	
	Women and Partner	1.16 (0.90,1.52)	
Age at First Sex	10 to 15 years	1	
	16 to 20 years	0.85 (0.60,1.18)	
	21 and 36 years	0.90 (0.50,1.31)	
Number of Children at First Use	No child	1	
	1 to 2 children	1.16 (0.82,1.63)	
	3 to 12 Children	1.17 (0.71,1.91)	
Husband FP Feeling	He disapprove/Not ok	1	
	He is_ OK_ with it	0.85 (0.97,1.09)	
Birth Order	No child	1	
	1 child	0.27 (0.15,0.50)***	
	2 Children	0.14 (0.07,0.29)***	
	3 children	0.11 (0.05,0.25)***	
	4 + children	0.09 (0.03,0.22)***	
Distance to Health Facility	< 1 km	1	
	1 to 2 km	0.93 (0.65,1.35)	
	2 km and above	0.72 (0.50,1.15)	
SDPfacility_type	Hospital	1	
	Health center	1.20 (0.60,2.50)	
	Health post	1.43 (0.65,3.16)	
	Health clinic	1.13 (0.52, 2.43)	
# of Provided in the Nearest SDP	Provide_4methods	1	
	Provide_5 + Methods	1.49 (1.01,2.19)*	1.34 (0.87,2.10)
Method Stock	No stock out	1	
	At least One Stock out	0.84 (0.63,1.12)	

*p value < 0.05 ** *p* value < 0.01 *** *p* value <0.001

^+^Only results for two independent variables presented in both outcome came variable categories since these two are only variables that violated the assumptions of ordinal logistic regression. For the rest of the variables, the assumption is fulfilled, and the estimates are the same across the two cumulative logits

The two variables namely, women who intended to have additional child and nearest SDP provide 5 or more contraceptive methods have disproportional association across the cumulative logit of being in the upper categories of women emotional fertility intention, leading asymmetrical cumulative logit. The association was presented separately for the two of these independent variables since it’s the same for all other variables which met the parallel lines assumption (Table 6).

Elder women, women who wanted to have another child and whose contraceptive use was decided by the health care provider had an increased symmetrical odds to be in one of the higher level categories of women emotional intentional feeling. Birth order were found to have proportional association across the categories, same with each cumulative logit, i.e. lowered cumulative probability of being in the higher level of women emotional fertility intention categories.

The odds of being in the higher level emotional fertility intention categories was 6.75 (3.11,14.62) times higher among elder women aged 35 to 49 compared with their younger counter parts. The finding showed that the type of contraceptive use decision maker affects the hierarchal women fertility intention, accordingly when the health care provider decided on women contraceptive use similar cumulative logit of 1.83 (1.03, 3.24) across the the higher level categories of women emotional fertility intention was observed (Table 6).

Another factor was marriage duration where women in the higher categories of women fertility intention had symmetrically increased odds with increased duration of marriage. Accordingly, Women who stayed in marriage for 10 and above years were 3.79 (1.72, 8.31) more likely to be in the mixed or happy category the unhappy category. On the contrary, birth order lowered the odds of women emotional fertility intention of being in the higher categories by 86% 0.14 (0.07, 0.29) for women who have two children while who had 4 + children had only 0.09 (0.03, 0.22) times lower odds of emotional fertility intention if they became pregnant (Table 6).

The two variables namely: fertility desire and nearest SDP provided 5 or more contraceptive methods have no similar association across the cumulative logit of being in the two high ranking categories of women emotional fertility intention, leading asymmetrical cumulative logit. Hence, the asymmetrical effect of fertility desire in the higher emotional fertility intention categories is as follows: those who wanted to have an additional child had a 3.16 (2.28, 4.36) higher odds to be in the mixed or happy category than being in unhappy. Further, this tendency was even stronger at higher categories of emotional fertility intention 4.83 (3.23, 7.23) (Table 6).

Similarly, among the group level nearest health facility related variables, the nearest health facility which provided 5 or more contraceptive methods was found to have had asymmetrical cumulative logit on women emotional fertility intention. The number of methods provided in the nearest SDP result in asymmetrical odds of women being in the higher categories. Accordingly, women whose nearest health facility provided 5 or more methods had a 49% 1.49 (1.01, 2.19) increased likelihood of being in the mixed or happy category than being in the unhappy category of the emotional fertility intention while the number of methods had no significant association with emotional fertility intention at higher cumulative logit 1.34 (0.87,2.10) (Table 6).

## Discussion

Only results for the two independent variables presented in both higher categories of women emotional fertility intention since these two independent variables are the only variables that violated the assumptions of ordered logistic regression. For the rest of the variables include in the model, the assumption was fulfilled, and the estimates are the same across the cumulative logits of the higher level categories of women emotional fertility intention.

In countries like Ethiopia where males dominance in household decision making in general and on fertility and reproduction decisions in particular has been culturally accepted and social constructed, women control over their emotional fertility intention has been considerably suboptimal. Hence, investigating the factors affecting the level of women emotional fertility intention if they became pregnant and identifying the factors contributing for this variation has paramount importance to control the rapid population growth and sustain a developmentaly sound population growth rate. In midst of the SDG period, measuring women emotional fertility intention as measure of their reproductive health right and decision making over their reproductive life is hoped to generate and provide an actionable evidence for the ministry and relevant actors to improve women decision making on their emotional fertility intention in particular and reproductive health right in general which in turn contributes for success SDG 1.

Due to the dearth of empirical evidence which exactly fit the research title the authors are forced to look for additional alternative evidence for discussion including findings from fertility desire studies. In addition to the conventional discussion write up methods, the authors also used an indirect method of discussion such us comparing the finding with accomplished goals stated in national and international relevant policy and program endorsed documents and targets such as the success and challenge of the Ethiopian health extension program, the national health sector transformation plans along with relevant international documents.

The finding revealed that one in 2 women emotionally felt that they would not be happy: very unhappy (24.61%) and sort of unhappy (24.12%) when asked how would you feel If they got pregnant now while 17.03% reported that they have felt mixed feeling. On contrary, one third would felt happy: 22.88% felt sort of happy, 11.36% felt very happy. This is partly attributed to the success of health extension program (HEP) in Ethiopia; the achievement of the Health Sector Transformational Plans I_II (HSTP 2) [23, 24] and the endorsement and implementation of the new reproductive health strategy running 2021 to 25 in which preconception care and fertility related targets were include [24].

To this end, the implication of the finding that only one in two women felt unhappy if they became pregnant indicated that we have a long way to go to make women decide over their fertility desire in general and the emotional fertility intention aspect in particular through availing preconception care in general and the family planning and fertility intention packages in particular [18, 26, 55]. Furthermore, strengthening the urban Health Extension Professional and addition of level IV Health extension workers in rural set up is likely to improve the quality care [56] at community level thereby enabling women to get informed counseling about their reproductive health needs and rights including planning and spacing their pregnancies and those who did that might planned their desired family size and felt happy if they learned they became pregnant since the pregnancy is likely to be intended. Moreover, the government’s effort to improve quality of maternal and child health care and respectful maternity care [57] along with the continuum of care is likely to encourage women to plan their pregnancies and subsequently to be happy if they became pregnant and the variation in community support for pregnant women to utilize the three domains of the maternal and new born continuum of care packages also contributes for this observed variation in the women emotional fertility feeling as revealed in a recent study on Pregnant Women Perceived Community Acceptance on Continuumof pregnant women’s perceived community acceptance of the continuum of maternal and newborn care and its Correlatesrelatives in Ethiopia: Community -based 2 -year follow -up study [58].

The variation in the degree of women emotional fertility intention might be related with the socially constructed and culturally accepted males dominance in matters that affect women including women reproductive autonomy and their fertility control among others in low and middle income countries including Ethiopia. A recent nationally representative study from Ethiopia on partner-perpetrated pregnancy coercion inhibits women’s reproductive autonomy reported that approximately 20% of Ethiopian women reported past-year pregnancy coercion (11.4% less severe; 8.6% more severe), ranging from 16% in Benishangul-Gumuz to 35% in Dire Dawa [2]. Addressing this gap in women emotional fertility intention and health would help to track the success of SDG gaol 5.6.1 [18] and SDG goal 1 and the reproductive health targets articulated in the current national reproductive health plan running from 2021 to 25 [26]. Poor patient-provider communication and inadequate support of women’s autonomy contributed most to poor person-centered maternity care as well contribute for this variation in the level of women emotional fertility intention as its well-articulated on a study from Ethiopian entitled: Understanding variation in person-centered maternity care: Results from a household survey of postpartum women in 6 regions of Ethiopia [1].

The finding that elders had increased likelihood of symmetrical cumulative odds across the higher level categories of women emotional fertility intention [59] is likely emanated from their previous pregnancy and child bearing experience leading them well prepared for their index pregnancies and as result of improved spousal communication [60].

It is found that women intended to have another child had an increased but disproportionate association with the higher level categories of women emotional fertility intention might be related with the families’ aspiration to achieve the desired family size and can be seen as exercising their reproductive right and reproductive autonomy [61]. Moreover, the finding that being in the higher level categories of women emotional fertility intention was lower among those with higher birth order might be related with women prior pregnancy experience [59] and is likely related with male decision making on the number of children as stated in [12, 62, 63] and women decided to limit the family size but being forced to be pregnant because of their husband and/or partner insisted to have more children. This is in line with findings on women fertility desire [40, 64].

The results showed that the nearest health facility which provided 5 or more contraceptive methods was found to have had asymmetrical cumulative logit of women emotional fertility intention might indicate the link between contraceptive access, pregnancy and child bearing [65]. It might also be related with contraception and fertility transition [66] as well as women fertility desire and contraceptive behavior might also be possible explanation [67].

The finding showed that the type of contraceptive use decision maker affects the hierarchal variation in women fertility intention, accordingly when the health care provider decided on women contraceptive use similar cumulative logit of across the categories of women emotional fertility intention was observed which had an implication of the poor quality counseling [68, 69] and we need a long way to go to empower women on the their contraceptive use [70, 71] and also might be likely related with poor patient-provider communication and inadequate support of women’s autonomy contributed most to poor person-centered maternity care [1].

This study is not spared of limitations. To begin with, data were not collected from Tigray Region due to the conflict, therefor any form of generalization need to consider this in mind. Moreover, though reliance on self-reported data and potential biases exist particularity on women emotional fertility intention; such findings offer meaningful insights that advance the field of study. However, further research is needed to validate and build upon these initial results. Furthermore, because of PMA 2020 didn’t collect information on variables such as husband desired number of children, husband employment and women employment were not measured in this study.

As a strength, this study addresses potential confounder variables, notable group level variable. This study included additional individual and community level variables including health facility related variables.

## Conclusion

Nearly one in two women 48.73% (95% CI: 46.21%, 51.23%) reported unhappy if they became pregnant while one in 6 17.03% (95%CI:15.26%, 18.96%) and one third felt mixed feeling and happy if they became pregnant respectively calling up on intended and spaced pregnancies by ensuring women reproductive and economic empowerment to empower women over their fertility.

Activities and efforts that promote intended, spaced pregnancies and diversifying access to contraceptive methods in the nearest health facilities are likely to improve women emotional fertility intention; and activities that enable women to decide their contraceptive use by their own as well. The finding that health care providers decides on contraceptive use call for activities to improve quality of contraceptive use counseling to enable women decide by the themselves while the access of diversified methods in the nearby health facility create an opportunity for women to get the method they wanted and make them emotionally well. These two activities are hoped to enable women to plan their fertility thereby increasing their emotional wellbeing. The activities and interventions need to be tailored across regions and need to be age sensitive.

The implication of the findings calls strengthening postpartum contraceptive counseling, installing the inter pregnancy preconception package in the health system, enhancing informed contraceptive use decision making and promoting longer and healthy marriage.

## Data Availability

The datasets generated during the study are publicly available from the PMA website. https://www.pmadata.org/data/request-access-datasets.

## Abbreviations

AOR: Adjusted Odds Ratio
CS: Cross Sectional
EA: Enumeration Areas
HH: Households
GOLR: Generalized Ordered Logistics Regression
PMA: Performance for Monitoring for Action Ethiopia
SDP: Service Delivery Point
SNNPR: The former Southern Nations, nationalities and Peoples Region
